# Editorial: Reviews in psychiatry 2023: personality disorders

**DOI:** 10.3389/fpsyt.2025.1567144

**Published:** 2025-02-26

**Authors:** Massimiliano Beghi, Michele Sanza

**Affiliations:** Department of Mental Health and Addictions, Azienda Unità Sanitaria Locale (AUSL) Romagna, Forlì-Cesena, Cesena, Italy

**Keywords:** oxytocine, personality, seizures, musculoskeletal disorders, dimension

## Introduction

In this Research Topic, we provide an overview and discussion of key points from the nine articles published in this 2022 Research Topic entitled “*Reviews in psychiatry 2023: personality disorders*.” The overview has been thematically organized by topic.

## The dimensions of personality

The dimensional view of personality disorders (PDs) represents these conditions as extreme variants of normal personality continua. Hualparuca-Oliveira et al. examined whether there is a sufficient correlation between PDs measures of the ICD-11 (1) and DSM 5 (2) alternative models of personality disorder (AMPD) in the general population. The quality of the sixteen included studies was moderate. The authors found a strong and significant degree of heterogeneity and moderate association both for the overall model (r=0.62) and for the subgroup of associations (0.57 for the severity model and 0.63 for the association between the ICD-11 and DSM 5 AMPD trait models). When considering specific trait domains the correlation was high (0.71) for negative affectivity and moderate for Detachment (0.59), Dissociality/Antagonism (0.55), and Disinhibition (0,68). The results indicate satisfactory empirical evidence for the interchangeable usefulness of these measures between the two models.

Szucs et al. carried out a meta-analysis of 48 studies on the association between personality and help-seeking (HS). Schizotypal and borderline PDs and neuroticism traits are likely to engage in mental healthcare despite negative general attitudes toward care seeking, while paranoid, schizoid and obsessive-compulsive PDs are related to both negative HS attitudes and behavior, despite the unfavorable long-term prognosis. Limited evidence has linked extraversion to social support seeking and conscientiousness to care-seeking behavior.

Meta-analyses on the Five Factor model confirmed modest associations between neuroticism and more negative attitudes toward seeking professional psychological help and between agreeableness and more positive attitudes toward seeking professional psychological help, but the latter is lost in a sensitivity analysis. HS behavior is negatively associated with reality weakness and cynicism, and positively associated with abasement and rigidity.

## The neurobiology of personality disorders

Zhou et al. used two sample Mendelian randomization (TSMR) (3) to evaluate the causal relationship between BPD and atrial fibrillation (AF), and found that genetically predicted BPD was associated with an increased risk of AF in both the fixed effects inverse variance weighted model (p=0.0031) and the random effects inverse variance weighted model (p=0.0394). The authors pointed to genetic evidence of a causal relationship between BPD and increased risk of AF through TSMR, with no causal relationship of AF to BPD risk, concluding that inflammation may be a mediating factor between BPD and AF.

di Giacomo et al., systematically reviewed the existing literature on the complex interplay between BPD and oxytocin, following the hypothesis that dysregulation of the oxytocin system may contribute to the emotional instability and interpersonal difficulties observed in individuals with BPD (4). In the 70 studies included in their qualitative analysis, the authors found that Oxytocin may influence attachment styles, parenting behaviors, and stress responses, particularly in individuals with a history of childhood trauma. The interaction between oxytocin, genetics, early life experiences, and environmental factors contributes to the complexity of BPD, while genetic variations in the oxytocin receptor gene may influence social and emotional abilities and contribute to the development of psychopathology. Moreover, early adverse experiences, such as childhood maltreatment, could alter oxytocin functioning and impact social cognition and emotional regulation. They concluded that there is evidence for both potential benefits for specific symptoms, such as social threat avoidance, and adverse effects on nonverbal behavior and mentalizing.

## Personality disorders and musculoskeletal disorders

Quirk et al. carried out a systematic review to evaluate the quality and extent of evidence for associations of PD with chronic back/neck/spine conditions, arthritis, fibromyalgia, and reduced bone mineral density in eleven population based studies. The association was strongest for cluster B PDs. In younger groups, any PD, schizoid PD, and obsessive compulsive PD were each associated with increased odds of arthritis. Similarly, people with arthritis had increased odds of several specific PDs (paranoid, schizoid, histrionic, antisocial, avoidant and obsessive compulsive). Spinal pain was associated with BP, while muscular pain but not fibromyalgia was associated with PD and women with cluster A, but not cluster B and C PDs had poorer bone health. The authors concluded the need for new evidence from case control and cohort studies.

## Personality disorders and seizures

People with temporal lobe epilepsy (TLE) have frequently been described as having specific personality features (hyper-religiosity, hypergraphia, hyposexuality, and irritability) called Geschwind syndrome (5). Viola et al., in a mini-review of 23 studies, tried to collect and synthesize the existing evidence on PDs in subjects with epilepsy. They found higher prevalence rates (18-42%) of PDs in focal epilepsies (not specifically TLE) that are candidates for surgery, with cluster C personality being the most common. In juvenile myoclonic epilepsy prevalence rates were 8-23% with no strong correlation with any subtype. In functional seizures (FS), the prevalence ranged between 30% and 60% with a higher correlation with cluster B PDs. Comorbidity with PDs complicated treatment. The authors pointed out that studies have usually focused on traits rather than specific PDs and that often a substantial gap in knowledge exists about the common etiology, and effects of AEDs and epilepsy surgery in PDs.

The role of personality in the development of FS has been studied in recent literature (6, 7). Sammarra et al. systematically reviewed 14 studies on the FS population (prevalence of PDs, frequency of clusters A, B and C and comparison with patients with epilepsy). The rate of PDs as comorbidity in FS ranged from 18% to 87%, with a mean of 53.7% (41.7%-84.6% cluster B, 0%-48.3% for cluster A and 7.7%-75.9% for cluster C) Individuals with FS have nearly three times the odds of having PDs than patients with epilepsy (p<0.00001), a fourfold increased OR of having cluster B, (p<0.00001), an OR<1 of having cluster A (p=0.024), or cluster C (p=0.014) PD. The authors concluded that future research should assess the advantages of a systematic evaluation of personality disorders in FS, to address specific treatment planning and evaluate its effectiveness on seizure recurrence, psychological comorbidities and quality of life.

## Personality traits and axis I spectrums

More than 25% of patients diagnosed with obsessive-compulsive disorder (OCD) do not respond adequately to treatment (8) and the complex examination of risk factors can be conducted by using a new approach in the study of OCD, namely the empirical and theoretical framework of maladaptive schemas. Csigò and colleagues tried to identify the early maladaptive schemas characteristic of 112 (58 men and 54 women) Hungarian patients diagnosed with OCD, and to examine the presence and severity of comorbid anxiety and depressive symptoms in light of early maladaptive schemas (Mistrust-Abuse, Inferiority/Shame, Dependence/Incompetence, Insufficient Self-Control/Self-Discipline and Entitlement/Grandiosity (reverse effect)). Not the severity, but the *number* of the early maladaptive schemas showed a stronger correlation with the OCD symptom variables. The authors concluded that the relationship between OCD symptom severity and personality impairment does not appear to be directly proportional. Moreover, they emphasized that OCD is only one and not the most serious consequence of personality damage, indicated by early maladaptive schemas.

A more comprehensive understanding of the co-occurrence of alcohol abuse and PDs appears to be necessary for several reasons. For instance, treatments for each of these conditions are more likely to fail in the presence of the other (9). Jarcuskova et al. examined in their cross sectional study the clinical characteristics of 80 adults with alcohol dependence syndrome, 35% of whom had comorbidity with antisocial personality disorder (APD). The authors found that patients with a comorbid APD were younger, had lower education levels, were more likely to be unemployed and unmarried, and started drinking earlier (p<0.05). They usually have traumatic experiences, and were more likely to report comorbid anxiety, depression stress, (p<0.05), sleep problems (P = 0.058), and linguistic and attention deficits (P = 0.046). The authors concluded that understanding the comorbidities could lead to targeted interventions.

## Conclusions

This Research Topic underscores the multifaceted nature of personality disorders (PDs) and their extensive implications for clinical practice and research. The studies reviewed reveal the intricate interplay between dimensional models of personality and categorical approaches, emphasizing the potential for harmonization between frameworks such as the ICD-11 and DSM-5. Furthermore, neurobiological insights, such as the role of genetic factors, inflammation, and neuropeptides such as oxytocin, illuminate the biological underpinnings of PDs, offering promising avenues for biomarker development and targeted interventions. The associations between PDs and physical health conditions, such as musculoskeletal disorders and epilepsy, highlight the bidirectional relationships between mental and physical health, necessitating integrated approaches to treatment. Similarly, the exploration of comorbidities, such as obsessive-compulsive disorder and alcohol dependence, illustrates how maladaptive schemas and personality traits add to the complexity of treatment, often requiring personalized and multidisciplinary strategies to address overlapping symptoms and improve outcomes. These studies also draw attention to the societal and behavioral dimensions of PDs, particularly about help-seeking behavior and the stigma surrounding mental health.

Future studies should aim to address existing gaps, particularly through longitudinal and cohort designs, to build a more comprehensive understanding of the mechanisms and impact of personality disorders across different populations. Emphasizing both biological and psychosocial determinants, future efforts should focus on early detection, comprehensive assessment, and tailored therapeutic approaches to address the complex needs of individuals with PDs. By bridging the gaps between research and practice, we can contribute to more holistic and effective care for this vulnerable population.
